# *lefser*: implementation of metagenomic biomarker discovery tool, *LEfSe*, in R

**DOI:** 10.1093/bioinformatics/btae707

**Published:** 2024-11-25

**Authors:** Asya Khleborodova, Samuel D Gamboa-Tuz, Marcel Ramos, Nicola Segata, Levi Waldron, Sehyun Oh

**Affiliations:** Institute for Implementation Science in Population Health, City University of New York School of Public Health, New York, NY 10027, United States; Department of Epidemiology and Biostatistics, City University of New York School of Public Health, New York, NY 10027, United States; Institute for Implementation Science in Population Health, City University of New York School of Public Health, New York, NY 10027, United States; Department of Epidemiology and Biostatistics, City University of New York School of Public Health, New York, NY 10027, United States; Institute for Implementation Science in Population Health, City University of New York School of Public Health, New York, NY 10027, United States; Department of Epidemiology and Biostatistics, City University of New York School of Public Health, New York, NY 10027, United States; Cellular, Computational and Integrative Biology, University of Trento, Trento, Provo 38123, Italy; European Institute of Oncology, Istituto di Ricovero e Cura a Carattere Scientifico, Milan 20139, Italy; Institute for Implementation Science in Population Health, City University of New York School of Public Health, New York, NY 10027, United States; Department of Epidemiology and Biostatistics, City University of New York School of Public Health, New York, NY 10027, United States; Institute for Implementation Science in Population Health, City University of New York School of Public Health, New York, NY 10027, United States; Department of Epidemiology and Biostatistics, City University of New York School of Public Health, New York, NY 10027, United States

## Abstract

**Summary:**

*LEfSe* is a widely used Python package and Galaxy module for metagenomic biomarker discovery and visualization, utilizing the Kruskal–Wallis test, Wilcoxon Rank-Sum test, and Linear Discriminant Analysis. R/Bioconductor provides a large collection of tools for metagenomic data analysis but has lacked an implementation of this widely used algorithm, hindering benchmarking against other tools and incorporation into R workflows. We present the *lefser* package to provide comparable functionality within the R/Bioconductor ecosystem of statistical analysis tools, with improvements to the original algorithm for performance, accuracy, and reproducibility. We benchmark the performance of *lefser* against the original algorithm using human and mouse metagenomic datasets.

**Availability and implementation:**

Our software, *lefser*, is distributed through the Bioconductor project (https://www.bioconductor.org/packages/release/bioc/html/lefser.html), and all the source code is available in the GitHub repository https://github.com/waldronlab/lefser.

## 1 Introduction

Linear discriminant analysis (*L*DA) *Ef*fect *S*iz*e* (*LEfSe)* is a Python-based software that combines statistical tests with biological consistency estimation to detect features significantly enriched in one or more investigator-defined groups (Segata *et al.* 2011). *LEfSe* has been extensively used in thousands of metagenomic studies, facilitating the identification and visualization of potential biomarkers associated with various conditions, including human diseases (Zeller *et al.* 2014), environmental perturbations (Thijs *et al.* 2017), and ecological processes (Mirete *et al.* 2016). Although numerous alternative algorithms have since been developed to identify and rank differentially abundant microbial features (Nearing *et al.* 2022), *LEfSe* remains a very common and popular approach: as of the time of writing, >40% of all microbiome studies (455 out of 1087) cataloged in *BugSigDB* used *LEfSe* (Geistlinger *et al.* 2024). The continued preference for this method >10 years after its original publication reflects the practicality of the software and the biological relevance of the results it produces.

Although the *LEfSe* Python and Galaxy interfaces are widely used, the algorithm has remained difficult to utilize for R/Bioconductor (Gentleman *et al.* 2004) users. Some steps of the *LEfSe* algorithm lack statistical justification and add unnecessary sampling error, providing an opportunity for improving the accuracy of biomarker detection. The *microbiomeMarker* (Cao *et al.* 2022), which integrates commonly used differential analysis methods and machine learning-based approaches, provides an R implementation but does not closely reproduce the *LEfSe* algorithm or implement its visualization capabilities.

To address these gaps, we developed the *lefser* R/Bioconductor package to implement the core functionalities of *LEfSe* for R-based microbiome analysis pipelines and benchmarking. *lefser* improves the efficiency and reproducibility of the original algorithm by replacing a needless bootstrap estimate of LDA coefficients with a more precise direct estimation and eliminating an unnecessary addition of small amounts of random noise that was used to avoid rank-deficient input data matrices. We benchmark *lefser* using human and murine microbiome datasets, highlighting its comparable and improved performance for statistical analysis and effect size visualization.

## 2 Methods

### 2.1 *lefser* algorithm

*lefser* identifies biomarkers between two primary conditions (classes) and optional sub-conditions (subclasses) from the relative abundances of features (e.g. microbial taxa) across samples. First, it performs a Kruskal–Wallis test, as implemented in the *stats* package (stats::kruskal.test) (Bolar 2019), to identify features that show statistically significant differential abundance between the classes defined by the classCol argument (the default significance threshold is α = 0.05). Only the features that reject the null hypothesis are further analyzed. If the subclass is specified through the subclassCol argument, *lefser* performs a Wilcoxon Rank-Sum Test, as implemented in the *coin* package (coin::wilcox_test) (Hothorn *et al.* 2008), between subclasses within each class (the default significance threshold is α = 0.05). The features that violate the null hypothesis and have the same direction/sign of z-scores in all pairwise comparisons, which is the “strict” version of the multiclass strategies in *LEfSe* (Segata *et al.* 2011), are considered biomarkers. These biomarkers are subjected to Linear Discriminant Analysis (LDA), as implemented in the *MASS* package (MASS::lda) (Venables and Ripley 2003). Unlike *LEfSe*, where LDA model fitting uses a subset of samples (randomly selected ⅔ of the total number of samples) for 30x bootstrap iteration by default, *lefser* uses the entire dataset for LDA model fitting. While bootstrap is useful for estimating confidence intervals, the mean of the bootstrap sampling distribution is only an estimate of the value from the entire dataset. Thus, bootstrap in *LEfSe* is not only unnecessary for estimating LDA coefficients, but using the mean from bootstrap samples introduces an error in the estimate of the coefficient compared to using the entire dataset, which is how *lefser* calculates LDA coefficients (Supplementary Figs S1 and S2). The effect size for each feature is calculated as the absolute difference between the LDA discriminant scores of the two classes, scaled by the unit-normalized LDA coefficients to prioritize features with the largest differences between the two classes. The sign of the effect size is used to indicate the direction of difference. The final results (LDA score) from *lefser* represent log 10 of the absolute effect size value following the sign of the raw effect size. Only the features with an LDA score greater than or equal to the specified lda.threshold (the default is 2) are returned.

For inputs with many features, such as functional profiles from *HUMAnN* (Beghini *et al.* 2021), *LEfSe* needs very low thresholds for the Kruskal–Wallis and Wilcoxon Rank-Sum tests to avoid tens of thousands of features being subjected to LDA. To solve this, *lefser* enables multiple hypothesis testing corrections for the Kruskal–Wallis and Wilcoxon Rank-Sum tests, as implemented in the *stats* package (stats::p.adjust) (Bolar 2019) through the method argument (the default is none).

### 2.2 Software for benchmarking

We used the docker container (*biobakery/lefser*, version 1.0.0) to run *LEfSe*. We used *microbiomeMarker* (version 1.11.0) (Cao *et al.* 2022), an R package implementing *LEfSe* functionality, and *lefser* (version 1.15.11) in R 4.4.1, following the default parameters unless specified. A random number seed was set for *microbiomeMarker*, which is unnecessary for *lefser*. *LEfSe* sets the random number seed internally. Analysis of the difference between putative true positives (TP) and putative false positives (FP) was carried out as described previously (Calgaro *et al.* 2020) using the *benchdamic* package (version 1.11.0).

### 2.3 Datasets for benchmarking

We used three metagenomic datasets to evaluate the *lefser* algorithm. The first dataset was the whole metagenomic shotgun sequencing (WMS) samples from colorectal cancer (CRC) patients (Zeller *et al.* 2014). We excluded the adenoma condition and analyzed classes (control versus CRC) and sub-classes (adult versus senior) with the default parameters. The sample sizes for each group were control-adult (*n* = 46), control-senior (*n* = 20), CRC-adult (*n* = 45), and CRC-senior (*n* = 46). Second, we used the HMP 16S gingival samples with biological growth truth (Ramos *et al*. 2022) to evaluate the algorithm's accuracy. We selected 230 samples from 115 patients, where both sub- and supra-gingival plaque samples were collected during the same visit. The body subsites (subgingival_plaque and supragingival_plaque) were set to be the classes with the default thresholds. The last dataset was a 16S rRNA amplicon sequencing dataset from 20 *T-bet^-/-^ x Rag^-/-^* mice and 10 control mice (Garrett *et al.* 2010, Veiga *et al.* 2010, Segata *et al.* 2011). We used two classes (*rag2* and *truc*) under the Genotype and set the Kruskal–Wallis test and LDA thresholds to 0.01 and 2, respectively.

## 3 Results

### 3.1 Implementation

The *lefser* package contains two main functions—lefser and lefserPlot. The lefser function identifies microbial features, such as taxa, that are significantly different between two or more biological conditions and provides the effect sizes of these differences, which can be used to prioritize the most important features for further investigation. This function accepts three main inputs. The first required input (relab argument) is in a *SummarizedExperiment* object format with an abundance matrix (features in rows and samples in columns) stored in the *assay* slot. The other two inputs are information on classes (classCol argument) and sub-classes (subclassCol argument, optional) to make the comparisons across. The classCol argument is a required input that should be stored in a *colData* slot of the *SummarizedExperiment* object (default named “CLASS”). This function returns a data frame with two columns—the name of the features as potential biomarkers (“features” column) and their LDA scores (“scores” column). The lefserPlot function takes the output from the lefser function or a data frame with the same column names (“features” and “scores”) and returns a barplot of the candidate biomarkers with their LDA scores.

The lefser function allows users to determine the statistical thresholds for the biomarker discovery process and the output to be returned. Lower thresholds for kruskal.threshold and wilcox.threshold allows the detection of biomarkers with smaller *P*-values, while a larger lda.threshold returns biomarkers with higher effect sizes in order of importance. The default values are set to 0.05 for both kruskal.threshold and wilcox.threshold, and 2.0 for lda.threshold. The default lda.threshold of 2.0 corresponds to an effect size of 100 in relative abundance terms. It is important to note that statistical significance (low *P*-value) does not necessarily equate to biological relevance, and nonsignificant results (higher *P*-value) can still represent important biological effects obscured by measurement error or small sample size. Therefore, interpretation of results should consider both statistical significance and effect size (Wasserstein *et al.* 2019).

### 3.2 Algorithm improvements

*LEfSe* used bootstrap only to calculate the mean of LDA coefficients in bootstrap samples. There is no statistical justification for doing this, and the sampling error introduced during this process yields lower accuracy coefficients that converge to the full dataset values for large numbers of bootstrap iterations. To demonstrate the instability of the *LEfSe* algorithm, we performed 20 different bootstrap experiments with 5–100 iterations, increasing by 5. These iterations identified 25 different OTUs corresponding to 11 taxa, of which only 17 OTUs and 10 taxa were identified in all 20 experiments (Supplementary Fig. S2A). We confirmed that when *LEfSe* is run with sufficiently large numbers of bootstrap samples, the LDA coefficients it returns converge to the *lefser* coefficients (Supplementary Fig. S2B).

To avoid the potential for rank-deficient input data matrices causing an error during LDA, the original *LEfSe* algorithm added small, normally distributed random numbers to all values. However, this approach has several drawbacks and potential issues. First, it does not fundamentally change the relationship between variables—the collinearity is just slightly masked. It also introduces unnecessary randomness, reducing the accuracy of biomarker ranking. Finally, *LEfSe* ensured reproducible random number generation by setting the random seed internally, which may result in an undesirable side-effect if embedded in a larger program relying on independent random number generation (such as simulations). Thus, we removed the introduction of random noise in *lefser* and instead rely on filtering of colinear features, which are generally those that are rarely or never observed (Supplementary Fig. S1).

Finally, *lefser* introduces an option for multiple hypothesis testing corrections.

### 3.3 Visualizations

**Figure 1.**
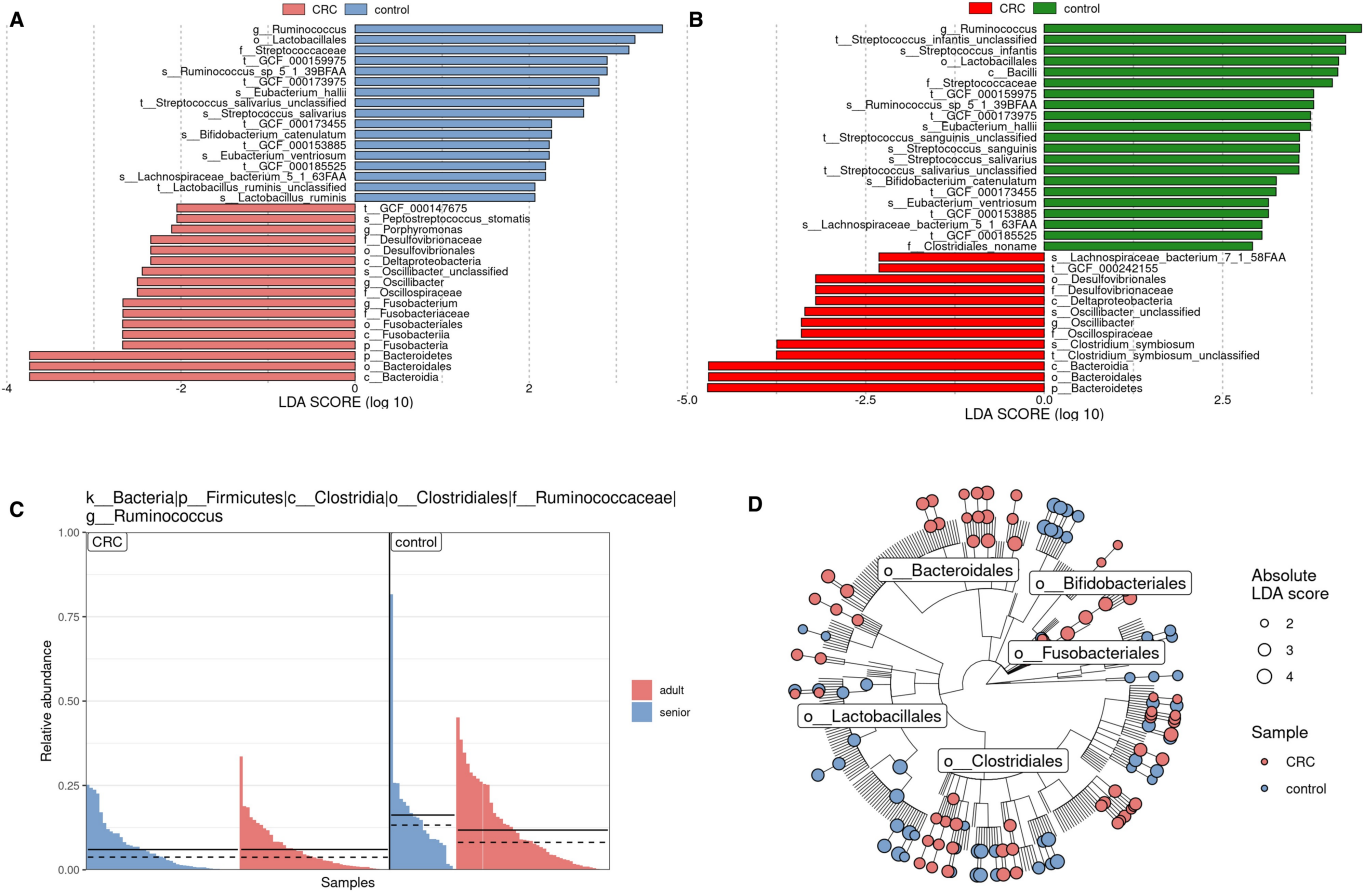
Comparison of biomarkers identified by *lefser* and *LEfSe* for CRC patients. We analyzed microbiome data from 157 participants (66 controls and 91 CRC cases). Both *lefser* and *LEfSe* were applied using default parameters, considering “*study_condition*” (control versus CRC) as the main class and “*age_category*” (adult versus senior) as the subclass. (A) and (B) show barplots of differentially abundant features identified by *lefser* and *LEfSe*, respectively, ranked by effect size. (C) The histogram displays the relative abundance of *Ruminococcus* [in the (0,1) interval] across classes (main panels) and sub-classes (color-coded). Solid and dashed lines represent mean and median abundances, respectively. (D) The cladogram illustrates taxonomic relationships of differentially abundant features between control and CRC groups. Node colors indicate the condition where taxa are more abundant, while node sizes reflect the absolute LDA scores. Users can choose the labels on tips and nodes.

*lefser* provides three publication-ready quality visualization options—the lefserPlot function displays LDA scores of the selected features as a barplot (Fig. 1A), the lefserPlotFeat plots the histogram of relative abundance [in the (0, 1) interval] of the selected feature (Fig. 1C) and the lefserPlotClad draws the cladogram of the significantly more abundance taxa and their LDA scores (Fig. 1D), providing a clear, hierarchical view of which bacterial groups are differentially abundant in classes. To improve accessibility, *lefser* uses a color-blind-friendly color palette as the default.

### 3.4 Benchmarking

We benchmarked *lefser* against *LEfSe* using three datasets. First, we analyzed a human shotgun metagenomic dataset collected to study colorectal cancer (CRC) (Zeller *et al.* 2014). We observed more similarity between *LEfSe* and *lefser* than with *microbiomeMarker* (Fig. 1A and Supplementary Fig. S3). We analyzed the taxa detected by *lefser* but not by *LEfSe* or vice versa and concluded that taxa identified by lefser but not *LEfSe* align better with current knowledge of microbial associations with CRC (Supplementary Results). Many taxa were identified only by *microbiomeMarker*, which implies potentially higher false positives.

Next, we analyzed the gingival dataset with known ground truth (Ramos *et al.* 2022). We compared the taxa annotated as aerobic, anaerobic, or facultative anaerobic in subgingival and supragingival plaque samples using *lefser*, *LEfSe*, and *microbiomeMarker*. All methods detected the anaerobic bacteria in the subgingival plaque with a strong statistical significance (Supplementary Fig. S4A). We evaluated the accuracy of the results by comparing them with the ground truth: putative true positives are (i) taxa annotated as aerobic and enriched in the supragingival plaque and (ii) taxa annotated as anaerobic and enriched in the subgingival plaque. We ranked the taxa based on their effect size and computed the difference between the putative true positives and the putative false positives across various threshold cutoffs (Calgaro *et al.* 2020, 2023). All methods were performed with similar accuracy (Supplementary Fig. S4B).

Lastly, we used the mice data previously analyzed by *LEfSe* (Segata *et al.* 2011) to investigate the fecal microbiota in a spontaneous colitis mouse model. Results from *lefser* and *LEfSe* were almost identical (Supplementary Fig. S5A and B), but a small difference was expected due to the use of random noise addition and bootstrap in the original implementation. This difference (i.e. two taxa detected by *LEfSe* but not by *lefser*) is also biologically less relevant based on the previous literature (Supplementary Results). From the same dataset, *microbiomeMarker* returned more taxa than the other two methods, implying a greater difference from *LEfSe* and potential false positives (Supplementary Fig. S5C and D).

## 4 Conclusions

We have implemented the *LEfSe* algorithm as the *lefser* package in Bioconductor and demonstrated its comparable and superior performance through benchmarking against the original Python and other R implementations. From the theoretical standpoint, *lefser* is a better methodology than *LEfSe*; *lefser* adopts the widely-used, well-verified algorithms (Kruskal–Wallis and Wilcoxon Rank-Sum tests) and makes it statistically more correct than *LEfSe* by removing two unnecessary sources of error—the bootstrap process, which introduced needless sampling variability, and the addition of random noise, originally intended to handle sparse input data but now better addressed through proper filtering. We demonstrated *lefser’*s algorithmic stability, capability to identify biologically relevant biomarkers, and higher agreement with another method. Additionally, *lefser* implements multiple hypothesis testing options, defaults a color-blind-friendly color schema for improved accessibility, and eliminates unintended side effects from internal random number seeding, which was used in the original *LEfSe*. In summary, *lefser* not only brings the popular *LEfSe* algorithm to the R/Bioconductor environment but also enhances its statistical accuracy, usability, and visualization capabilities.

## Supplementary Material

btae707_Supplementary_Data

## Data Availability

All the scripts to reproduce benchmarking examples and figures are available in the GitHub repository https://github.com/shbrief/lefserBenchmarking.
