# Supplementary material for: lefser: implementation of metagenomic biomarker discovery tool, LEfSe, in R
Source: Bioinformatics. 2024 Nov 25;40(12):btae707. doi: 10.1093/bioinformatics/btae707 (PMC11665633; doi:10.1093/bioinformatics/btae707)
Supplement: btae707_Supplementary_Data [file btae707_supplementary_data.pdf]

Supplementary Results

Compare features identified by different software

We compared the biomarkers identified by *lefser* and *LEfSe* from CRC patients' samples (**Figure 1 and Supplementary Figure 3**). We collected the discrepancies between the features identified by *lefser* versus *LEfSe*, and investigated their biological relevance and interpretability at the highest rank, which are twelve listed below:

- 5 by-lefser-only:
  - *Fusobacteria* group (5 entries)
  - *Porphyromonas*
  - *Peptostreptococcus stomatis*
  - GCF_000147675 (specific strain)
  - *Lactobacillus ruminis* group (2 entries)
- 7 by-LEfSe-only:
  - *Clostridium symbiosum* group (2 entries)
  - *Streptococcus sanguinis* group (2 entries)
  - *Streptococcus infantis* group (2 entries)
  - *Bacilli* (class level)
  - *Lachnospiraceae bacterium* 7_1_58FAA
  - *Clostridiales* (family level)
  - GCF_000242155 (specific strain)

Since *lefser* does not introduce unnecessary randomness present in the original *LEfSe* algorithm (sampling error through the bootstrap and addition of random noise to relative abundance data), we hypothesize that the taxa identified only by *lefser* should be more biologically relevant than those identified only by *LEfSe*:

lefser but not *LEfSe*:

- *Fusobacteria*: *Fusobacteria*, particularly *Fusobacterium nucleatum*, have been strongly associated with colorectal cancer[1–5]. Numerous studies have shown that *F. nucleatum* is enriched in CRC tissues and may play a role in tumor progression. The fact that *lefser*, but not *LEfSe*, detected *Fusobacteria* implies that *lefser*'s performance/sensitivity is potentially better than *LEfSe*.
- *Porphyromonas*: Some studies have also linked *Porphyromonas* to CRC[6,7]. While not as well-established as *Fusobacterium*, its presence might be biologically accurate.
- *Peptostreptococcus*: *Peptostreptococcus stomatis* has been associated with CRC in some studies[8–10].
- *Lactobacillus ruminis*: Some *Lactobacillus* species are often considered beneficial. Thus, this might need further investigation[11].

LEfSe but not *lefser*:

- *Clostridium symbiosum*: Recent studies have suggested that *C. symbiosum* could be a potential marker for early-stage CRC[12–14].
- *Streptococcus* species: The role of *Streptococcus* in CRC is less clear, but some studies have found associations. Missing these might or might not be critical[15].
- *Bacilli*: *Bacilli's role* in CRC is complex and not as straightforward as that of some other bacterial groups, like *Fusobacteria*. Some studies have found associations between certain *Bacilli* and CRC, while others have found protective effects[16,17].
- *Lachnospiraceae*: Some members of the *Lachnospiraceae* family have been rather associated with protection against CRC[18,19].
- *Clostridiales*: This order contains both potentially harmful and beneficial bacteria in the context of CRC[20,21].

Overall, biomarkers identified by *lefser* but not *LEfSe* align better with current knowledge of microbial associations with CRC, as most of the features detected by *lefser*-only are strongly or somewhat linked to CRC, while features detected by *LEfSe*-only are controversial or reduced in CRC. This confirms our expectation that lefser should more precisely identify biologically relevant biomarkers than *LEfSe* because it eliminates unnecessary introduction of noise and sampling error.

We also investigated the biological interpretability of the biomarkers identified by *lefser* and *LEfSer* from a spontaneous colitis mouse model (**Supplementary Figure 5**). Compared to *lefser*, *LEfSe* detected two additional taxa, genus *Papillibacter* and order *Bifidobacteriales*; their relationship with colitis is complex and has not been shown to be causative of spontaneous colitis, implying a weaker biological relevance. In contrast, the taxa detected by both *lefser* and *LEfSe* represent an increase in pro-inflammatory bacteria (*Bacterioidetes* and *Proteobacteria* species), alternations in butyrate-producing bacteria (specific *Firmicutes* species), and changes in the abundance of typically beneficial bacteria (*Bifidobacterium* species), all of which were previously identified as CRC-associated dysbiosis, potentially contributing to a pro-inflammatory environment and altered metabolic activities in the colon[22–26]. Thus, *lefser* and *LEfSe* strongly agreed on the biologically relevant, potentially correct biomarkers, and *lefser* did not miss any critical biomarkers that *LEfSe* can detect.

Supplementary Figures

Supplementary Figure 1. Biomarkers identified by the *lefser* with or without randomness. We analyzed microbiome data from 157 participants (66 controls and 91 CRC cases)[27] using *lefser* **(A)** with (the original *LEfSe* algorithm) or **(B)** without the source of randomness - perturbation and bootstrap. The same biomarkers were identified from both examples, except for two additional biomarkers (*Porphyromonas_asaccharolytica_unclassified* taxon and *Porphyromonas_asaccharolytica* species, whose association with CRC is not well established) from the 'With Randomness' algorithm. The difference in LDA scores between the two algorithms was negligible (mean = 0.09, standard deviation = 0.14) for absolute values of LDA coefficients ranging from 2.05 to 3.76.

A.

With Randomness

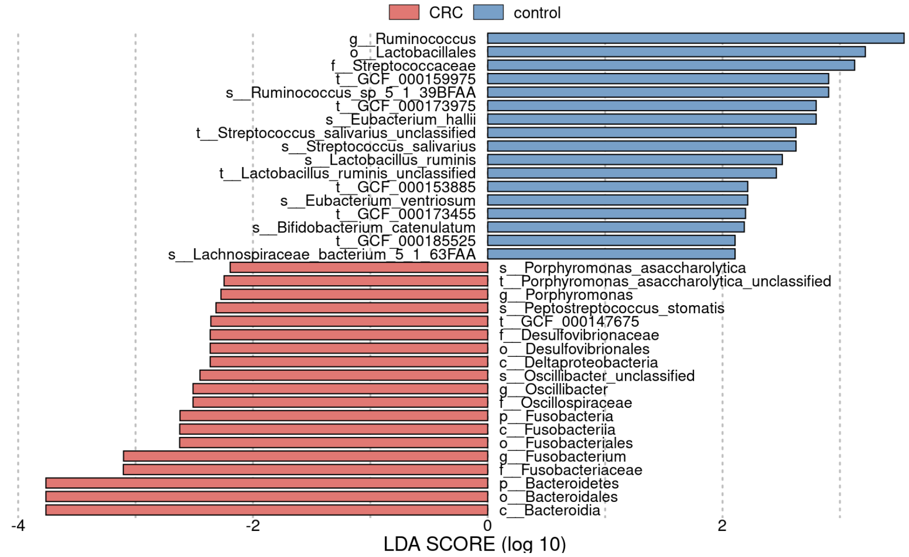

B.

Without Randomness

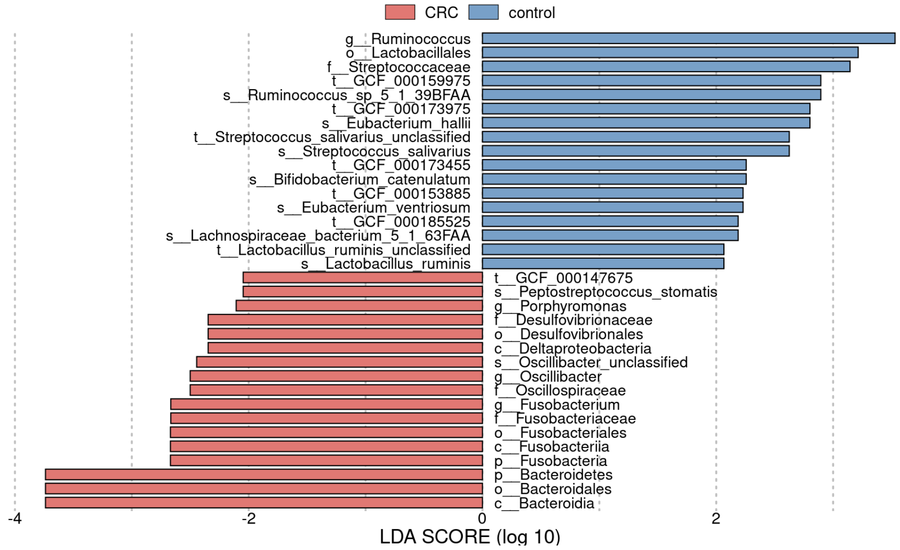

Supplementary Figure 2. Bootstrap in *LEfSe* makes the algorithm unstable. We analyzed the gingival dataset used in Supplementary Figure 4 was analyzed by *LEfSe* with more strict thresholds (Kruskal-Wallis test for 0.01 and LDA for 3) for 20 bootstrap iterations (from 5 to 100, increasing by 5). Twenty-five unique taxa were detected from these bootstrap iterations, where eight of them were not detected every time. **(A)** LDA scores from the 17 taxa detected at all times were stabilized with more bootstrap iterations. **(B)** LDA scores from the three different numbers of bootstrap iterations (5 (red), 30 (orange), and 100 (yellow)) were compared to those from *lefser* (blue).

A.

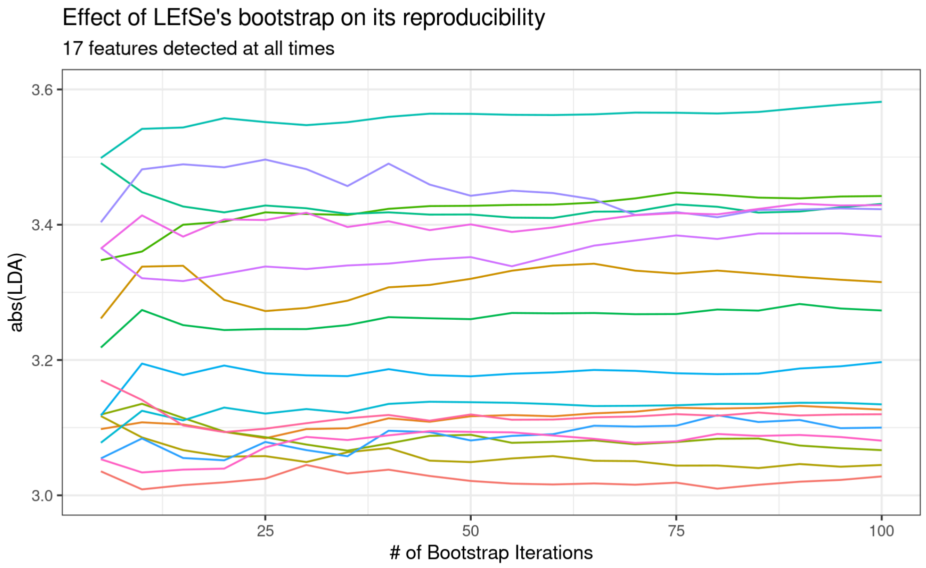

B.

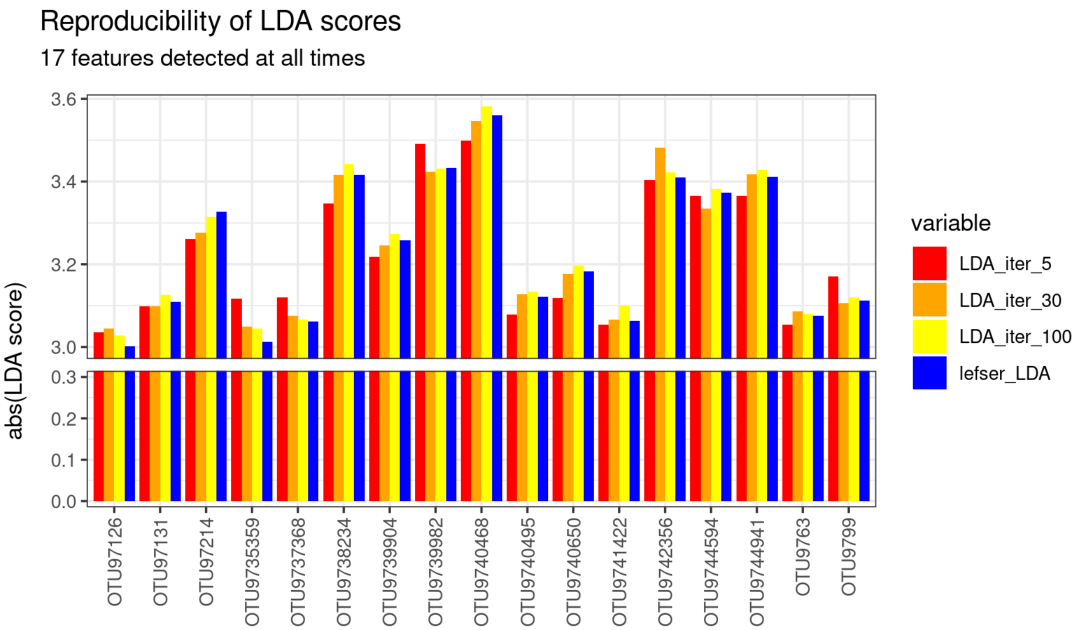

Supplementary Figure 3. Biomarkers identified by *lefser*, *LEfSe*, and *microbiomeMarker* for CRC patients. CRC patients' microbiome data from the Zeller14 dataset was analyzed using **(A)** *microbiomeMarker* (MM) with identical parameters as in Figure 1. **(B)** The Venn diagram shows the overlaps between biomarkers identified by each method.

A.

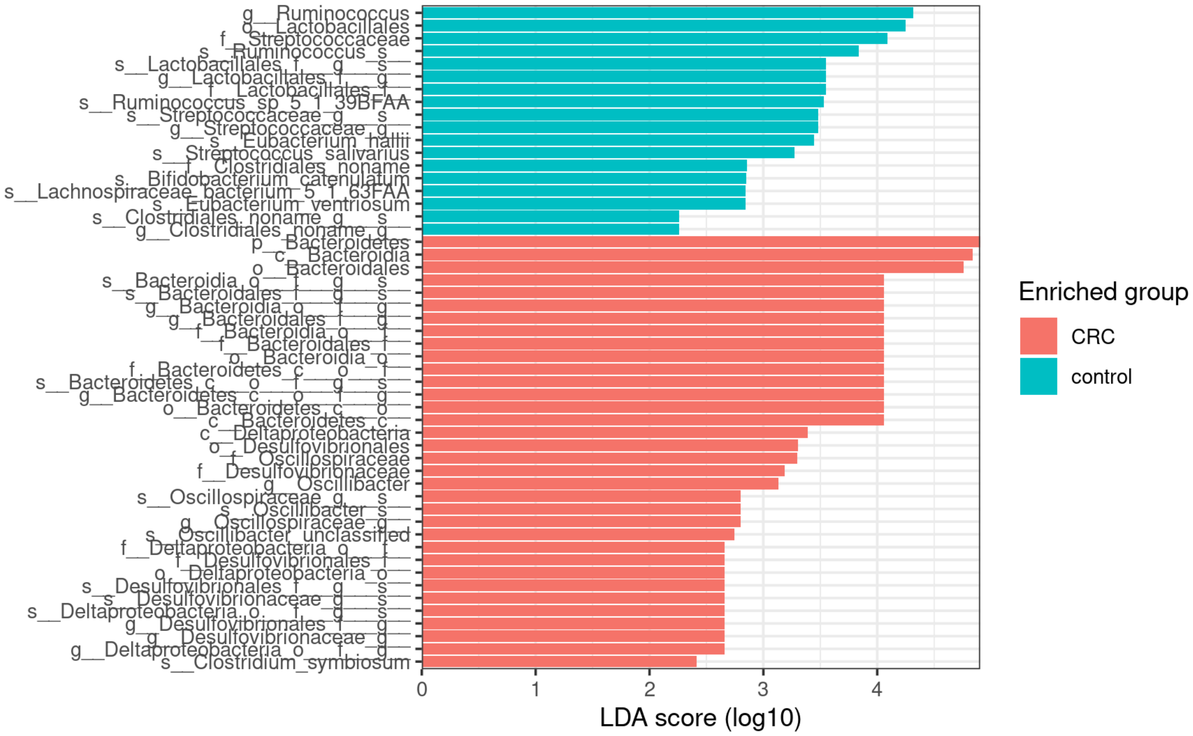

B.

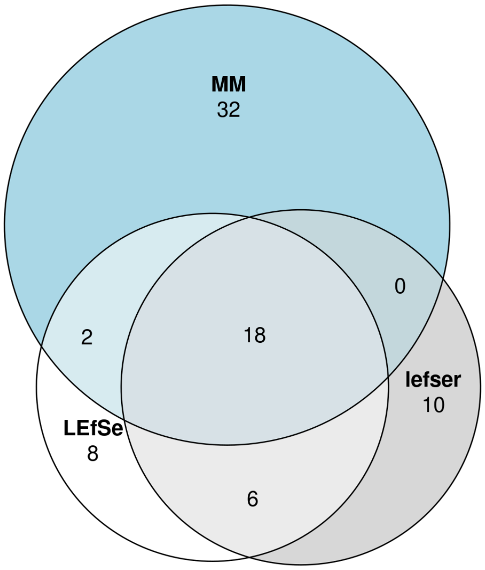

Supplementary Figure 4. Benchmark three methods using the microbiome dataset with the biological ground truth. We applied *lefser*, *LEfSe*, and *microbiomeMarker* on the subgingival and supragingival plaque samples. **(A)** Enrichment analysis of selected taxa annotated as aerobic (red), anaerobic (green), or facultative anaerobic (blue) in subgingival (left) and supragingival (right) samples. Bars indicate the number of differentially abundant taxa, defined with an FDR <= 0.1. Asterisks on top of the bars represent the p-value thresholds of the enrichment test (hypergeometric): * p-value <= 0.05, ** p-value <= 0.01, *** p-value <= 0.001. **(B)** Difference between putative true positives (TP) and putative false positives (FP) at different thresholds. The thresholds represent the top percent ranked taxa, using the effect size as a reference for the ranking (regardless of statistical test significance). Putative TP = The direction of the effect size corresponds to the expected taxon annotation based on the biological truth of the sample; Putative FP = The effect size direction does not correspond to the expected taxon annotation based on the biological truth of the sample.

A.

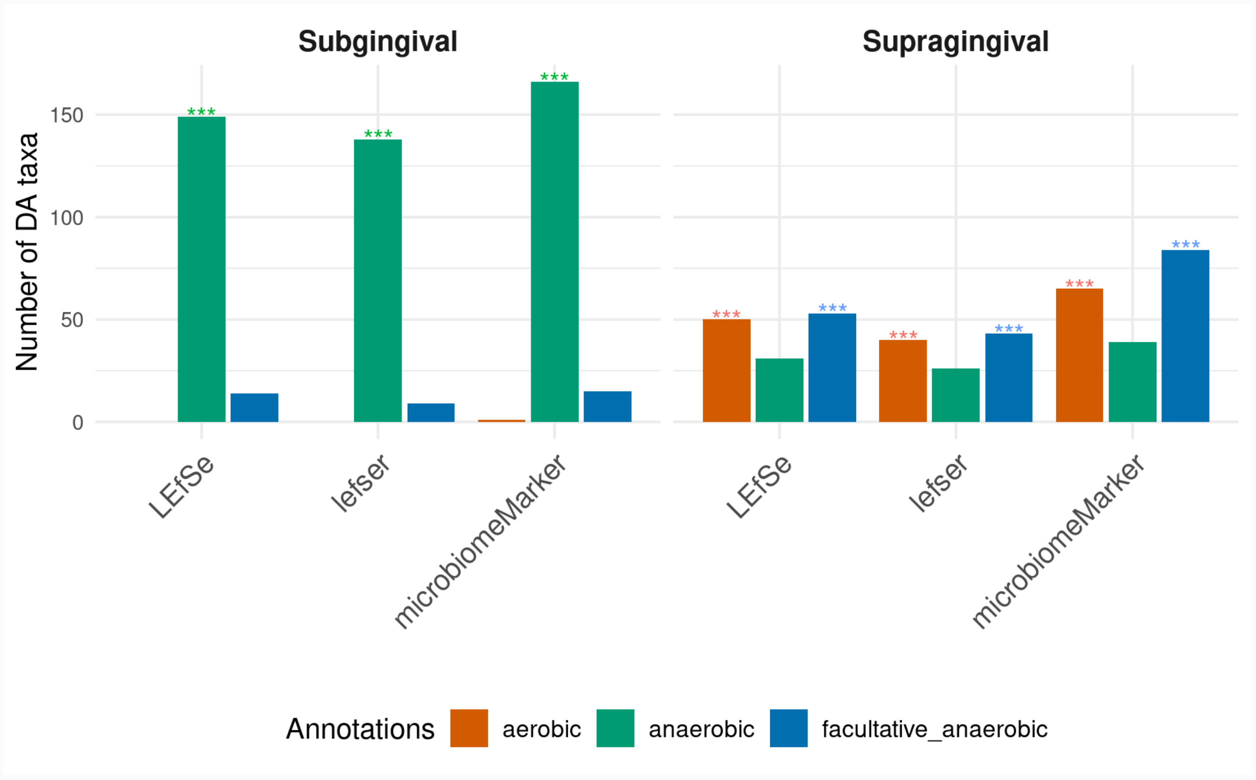

B.

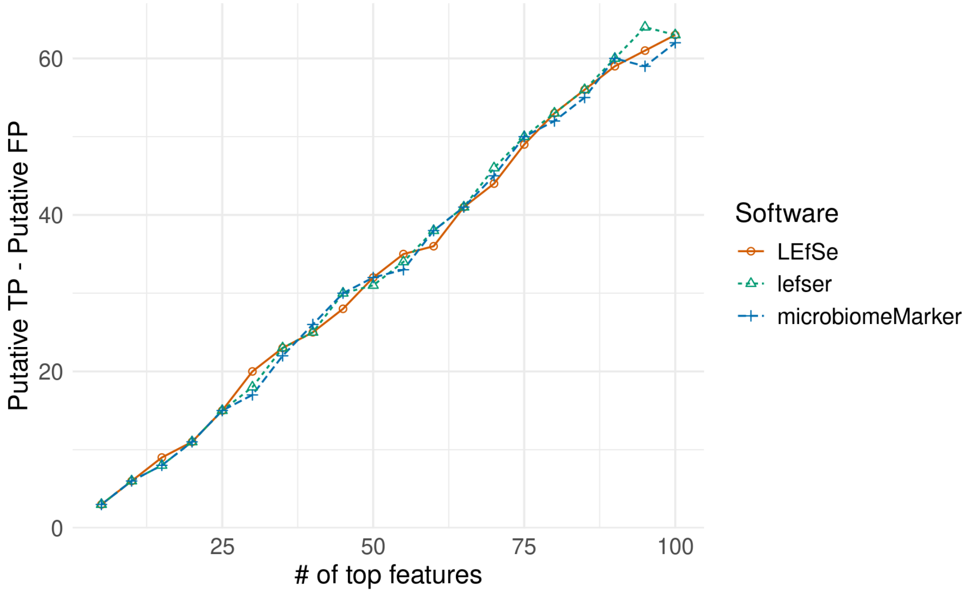
Putative TP - Putative FP
0
20
40
60
25
50
75
100
# of top features
Software
LEfSe
lefser
microbiomeMarker

Supplementary Figure 5. Comparison of microbial biomarker discovery results between *lefser*, *LEfSe*, and *microbiomeMarker* for mice dataset. Results from **(A)** *lefser*, **(B)** *LEfSe*, and **(C)** *microbiomeMarker* agreed with previous studies on the under-abundance of *Bifidobacterium* species in *T-bet*$^{-/-}$ *x Rag2*$^{-/-}$ (truc, case) mice compared to *Rag2*$^{-/-}$ (rag2, control) mice. All of them also identified the same seven abundant and twelve under-abundant species. *Bifidobacteriales* and *Papillibacter* were identified only by *LEfSe* but not by *lefser*, highlighting that exact reproduction is impossible due to *LEfSe*'s random sampling step. **(D)** The Venn diagram shows the overlaps between biomarkers identified by each method.

A.

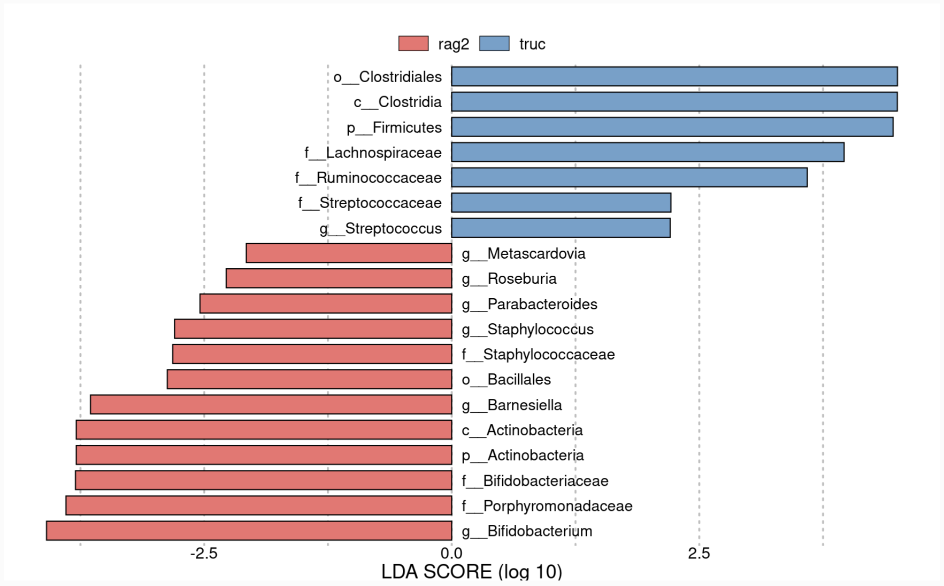

B.

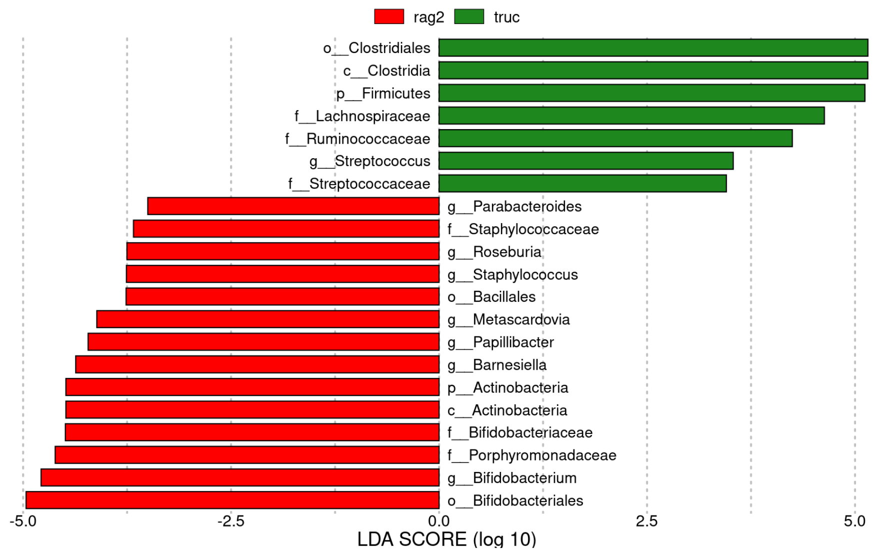

C.

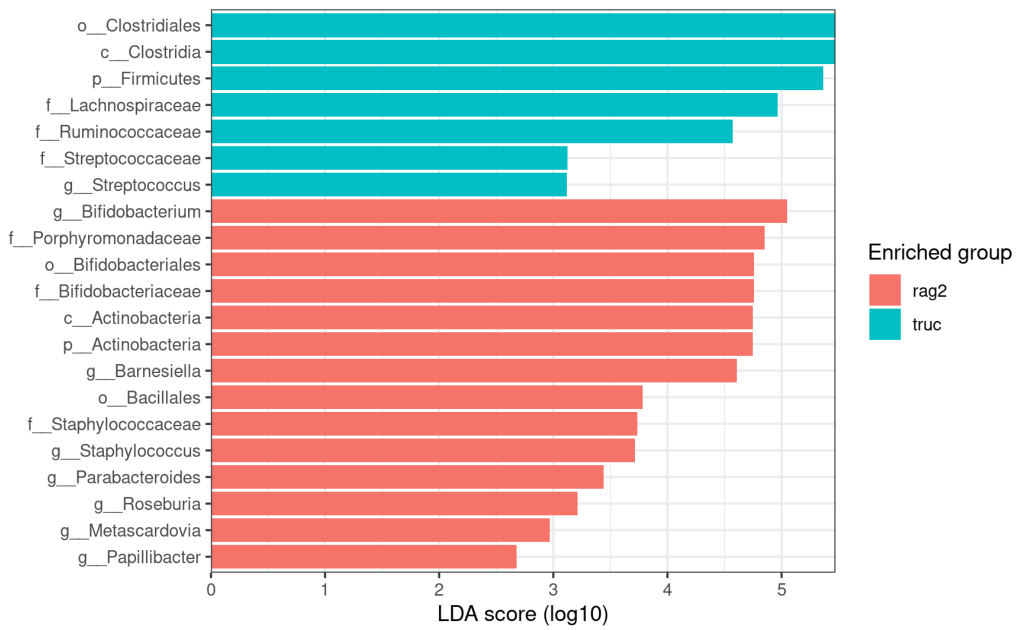
o__Clostridiales
c__Clostridia
p__Firmicutes
f__Lachnospiraceae
f__Ruminococcaceae
f__Streptococcaceae
g__Streptococcus
g__Bifidobacterium
f__Porphyromonadaceae
o__Bifidobacteriales
f__Bifidobacteriaceae
c__Actinobacteria
p__Actinobacteria
g__Barnesiella
o__Bacillales
f__Staphylococcaceae
g__Staphylococcus
g__Parabacteroides
g__Roseburia
g__Metascardovia
g__Papillibacter
0
1
2
3
4
5
LDA score (log10)
Enriched group
rag2
truc

D.

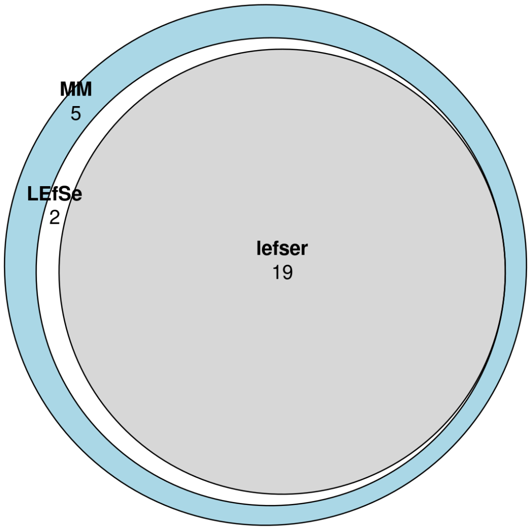
MM
5
LEfSe
2
lefser
19

Supplementary References

1. Bullman, S. *et al.* Analysis of Fusobacterium persistence and antibiotic response in colorectal cancer. *Science* **358**, 1443–1448 (2017).
2. Wang, N. & Fang, J.-Y. Fusobacterium nucleatum, a key pathogenic factor and microbial biomarker for colorectal cancer. *Trends Microbiol.* **31**, 159–172 (2023).
3. Castellarin, M. *et al.* Fusobacterium nucleatum infection is prevalent in human colorectal carcinoma. *Genome Res.* **22**, 299–306 (2012).
4. Kostic, A. D. *et al.* Genomic analysis identifies association of Fusobacterium with colorectal carcinoma. *Genome Res.* **22**, 292–298 (2012).
5. Brennan, C. A. & Garrett, W. S. Fusobacterium nucleatum - symbiont, opportunist and oncobacterium. *Nat. Rev. Microbiol.* **17**, 156–166 (2019).
6. Kerdreux, M. *et al.* Porphyromonas gingivalis in Colorectal Cancer and its Association to Patient Prognosis. *J. Cancer* **14**, 1479–1485 (2023).
7. Woo, B. H. *et al.* Oral cancer cells sustainedly infected with Porphyromonas gingivalis exhibit resistance to Taxol and have higher metastatic potential. *Oncotarget* **8**, 46981–46992 (2017).
8. Osman, M. A. *et al.* Parvimonas micra, Peptostreptococcus stomatis, Fusobacterium nucleatum and Akkermansia muciniphila as a four-bacteria biomarker panel of colorectal cancer. *Sci. Rep.* **11**, 2925 (2021).
9. Tsoi, H. *et al.* Peptostreptococcus anaerobius induces intracellular cholesterol biosynthesis in colon cells to induce proliferation and causes dysplasia in mice. *Gastroenterology* **152**, 1419–1433.e5 (2017).
10. Long, X. *et al.* Peptostreptococcus anaerobius promotes colorectal carcinogenesis and modulates tumour immunity. *Nat. Microbiol.* **4**, 2319–2330 (2019).
11. Colautti, A., Arnoldi, M., Comi, G. & Iacumin, L. Antibiotic resistance and virulence factors in lactobacilli: something to carefully consider. *Food Microbiol.* **103**, 103934 (2022).
12. Xie, Y.-H. *et al.* Fecal Clostridium symbiosum for noninvasive detection of early and advanced colorectal cancer: Test and validation studies. *EBioMedicine* **25**, 32–40 (2017).
13. Olovo, C. V., Huang, X., Zheng, X. & Xu, M. Faecal microbial biomarkers in early diagnosis of colorectal cancer. *J. Cell. Mol. Med.* **25**, 10783–10797 (2021).
14. John Kenneth, M. *et al.* Diet-mediated gut microbial community modulation and signature metabolites as potential biomarkers for early diagnosis, prognosis, prevention and stage-specific treatment of colorectal cancer. *J. Advert. Res.* **52**, 45–57 (2023).
15. Agnes, A. *et al.* Association between colorectal cancer and Streptococcus gallolyticus subsp. pasteuranus (former S. bovis) endocarditis: clinical relevance and cues for microbiota science. Case report and review of the literature. *Eur. Rev. Med. Pharmacol. Sci.* **25**, 480–486 (2021).
16. de Almeida, C. V., Taddei, A. & Amedei, A. The controversial role of Enterococcus faecalis in colorectal cancer. *Therap. Adv. Gastroenterol.* **11**, 1756284818783606 (2018).
17. Li, S. *et al.* Tumorigenic bacteria in colorectal cancer: mechanisms and treatments. *Cancer Biol. Med.* **18**, 0–0 (2021).
18. Zhang, X. *et al.* Tissue-resident Lachnospiraceae family bacteria protect against colorectal carcinogenesis by promoting tumor immune surveillance. *Cell Host Microbe* **31**,

418–432.e8 (2023).
19. Almeida, A. S. *et al.* Fiber-associated*Lachnospiraceae*reduce colon tumorigenesis by modulation of the tumor-immune microenvironment. *bioRxiv* (2021) doi:10.1101/2021.02.24.432654.
20. Mohammadi, M., Mirzaei, H. & Motallebi, M. The role of anaerobic bacteria in the development and prevention of colorectal cancer: A review study. *Anaerobe* **73**, 102501 (2022).
21. Dahmus, J. D., Kotler, D. L., Kastenberg, D. M. & Kistler, C. A. The gut microbiome and colorectal cancer: a review of bacterial pathogenesis. *J. Gastrointest. Oncol.* **9**, 769–777 (2018).
22. Borges-Canha, M., Portela-Cidade, J. P., Dinis-Ribeiro, M., Leite-Moreira, A. F. & Pimentel-Nunes, P. Role of colonic microbiota in colorectal carcinogenesis: a systematic review. *Rev. Esp. Enferm. Dig.* **107**, 659–671 (2015).
23. Zhu, Q. *et al.* Analysis of the intestinal lumen microbiota in an animal model of colorectal cancer. *PLoS One* **9**, e90849 (2014).
24. Chen, W., Liu, F., Ling, Z., Tong, X. & Xiang, C. Human intestinal lumen and mucosa-associated microbiota in patients with colorectal cancer. *PLoS One* **7**, e39743 (2012).
25. Quaglio, A. E. V., Grillo, T. G., De Oliveira, E. C. S., Di Stasi, L. C. & Sassaki, L. Y. Gut microbiota, inflammatory bowel disease and colorectal cancer. *World J. Gastroenterol.* **28**, 4053–4060 (2022).
26. Lucas, C., Barnich, N. & Nguyen, H. T. T. Microbiota, inflammation and colorectal cancer. *Int. J. Mol. Sci.* **18**, (2017).
27. Zeller, G. *et al.* Potential of fecal microbiota for early-stage detection of colorectal cancer. *Mol. Syst. Biol.* **10**, 766 (2014).
